# Detection rate of diabetic macular microaneurysms comparing dye-based angiography and optical coherence tomography angiography

**DOI:** 10.1038/s41598-020-73516-z

**Published:** 2020-10-01

**Authors:** Martin Stattin, Anna-Maria Haas, Daniel Ahmed, Ulrike Stolba, Alexandra Graf, Katharina Krepler, Siamak Ansari-Shahrezaei

**Affiliations:** 1 Karl Landsteiner Institute for Retinal Research and Imaging, Vienna, Austria; 2 Department of Ophthalmology, Rudolf Foundation Hospital, Juchgasse 25, 1030 Vienna, Austria; 3 Center for Medical Statistic, Informatics, and Intelligent Systems, Medical University of Vienna, Spitalgasse 23, 1090 Vienna, Austria; 4 Department of Ophthalmology, Medical University of Graz, Auenbruggerplatz 1, 8036 Graz, Austria

**Keywords:** Eye diseases, Diagnostic markers

## Abstract

Diabetic maculopathy (DM) is a microvascular dysfunction clinically characterized by microaneurysms (MA) leading to edema and central visual deprivation. This prospective explorative study investigated 27 eyes of 17 patients with DM by fluorescein/indocyanine green angiography (FA/ICGA; SPECTRALIS HRA-OCT, Heidelberg Engineering) and by swept source-optical coherence tomography angiography (SS-OCTA; DRI-OCT Triton Plus, Topcon) to identify clinically relevant MAs. The SS-OCTA cubes were split into the superficial capillary plexus (SCP) and the deep capillary plexus (DCP) according to the automated segmentation. The images of all modalities were superimposed for alignment by an Early Treatment Diabetic Retinopathy Study grid overlay and compared to each other. In total, the mean number of MAs in FA was 33.4 ± 22 (standard deviation) (median 27.5 [q1:21.75;q3:38.25]), in ICGA 24.9 ± 16.9 (17.5 [14;35]), in the SCP 6.5 ± 3.7 (5.5 [3.75;9.25]) and in the DCP 18.1 ± 10.5 (18.5 [10.75;23.5]). Mixed effects models between ICGA and the DCP were borderline significant (*p* = 0.048; 95% confidence interval 0.21 to 13.49), whereas all other imaging methods differed significantly. Quantitative analysis of MAs in DM showed a plausible agreement between ICGA and the DCP in SS-OCTA. These findings contribute to the imaging methodology in DM.

## Introduction

Diabetic maculopathy (DM) is a major cause of vision loss in developed countries^1–3^. Clinically significant macula edema (CSME) is an expression of fluid accumulation in a predefined area within the macula impairing the central vision^4^. The fluid derives from a breakdown of the inner blood–retina barrier, namely protrusions of the capillary walls or so called microaneurysms (MA)^5^. These outpouchings of capillaries are typically the earliest clinically visible signs of retinal damage and a hallmark of DM^2^.The exact identification of MAs is important as their location and number yields prognostic information on the severity of DM as well as on the clinical relevance for treatment guidance of focal or diffuse edema^6^.

Fluorescein angiography (FA) has been the gold standard to examine the retinal vasculature and graduate DM^7–9^. It is highly sensitive as it is able to visualize even smallest hyperfluorescent dots. However, it represents all vascularized structures of the fundus in an en-face image with obscuration of deeper layers in case of extensive leakage even in early phases such as present in diffuse edema. Therefore, its application for clinical management might not be favorable at all stages of the disease. Indocyanine green (ICG) has a higher molecular weight (775 g/mol) and binds to different transport proteins in the blood circulation with higher capacity and a tendency to form aggregates in solvents without extravasation^10^. The attributes of ICG angiography (ICGA) could be preferential for the quantification of clinically relevant MAs in DM and the planning of individual treatment algorithms^11–13^. Undesirable adverse events like nausea and emesis or even rare but serious anaphylaxis are addressed to the use of intravenous dye.

Optical coherence tomography angiography (OCTA) is a non-invasive dye-independent imaging technique based on the principle of motion contrast: the decorrelation of an OCT signal amplitude due to red blood cell movement^14,15^. It enables the visualization of flow signals at several distinct layers namely the superficial capillary plexus (SCP), the intermediate capillary plexus (ICP), the deep capillary plexus (DCP) or the choriocapillaris. Numerous studies investigated the potential of OCTA technology in the axial detection of macular MAs preferentially located in the DCP, where CSME almost exclusively originates^16–20^.

In the light of the above, we evaluated the quantitative appearance of macular MAs based on their clinical manifestation according to the Early Treatment Diabetes Retinopathy Study (ETDRS) grid in dye-based angiography and compared the results with those observed in the SCP and the DCP imaged by swept source (SS)-OCTA.

## Results

### Study participants

For this prospective explorative study 27 treatment-naive eyes of 17 diabetic patients were recruited from our tertiary eye care center (Medical Retina Unit, Department of Ophthalmology; Rudolf Foundation Hospital Vienna; Karl Landsteiner Institute for Retinal Research and Imaging) with a male preponderance (59%). The median age at the time of recruitment was 58 (range 36–76) years. Both eyes of 10 patients and 7 sole eyes (86% right eyes) were included. All patients underwent a complete ophthalmic examination including best-corrected visual acuity (BCVA) using the ETDRS charts at 4 m—counting every correctly read letter—and converted to Snellen. BCVA was 20/32 + 1 (0.92 ± 0.3 ETDRS) on average (range 0.06–1.26 ETDRS). Eyes with a funduscopic appearance of DM and diabetic retinopathy (DR) ranging from mild non-proliferative (NPDR) to proliferative (PDR) changes without the urgent need of surgical intervention were included. Mild to severe NPDR was present in 22 eyes with a CSME evident in 11 eyes, leaving 5 eyes with PDR. In addition to routine fluorescein angiography (FA), indocyanine green angiography (ICGA) and swept source-optical coherence tomography angiography (SS-OCTA) split into the superficial capillary plexus (SCP) and the deep capillary plexus (DCP) by automated segmentation were conducted. Exclusion criteria included previous treatment for DM or DR independent of cataract surgery and bad image quality due to motion artefacts in the cases of opaque media or fixation error. In total, 9 (33%) eyes have already had successful cataract surgery, while 10 (37%) eyes showed cataract formation to some degree.

### Image grading and MA counting

**Figure 1:**
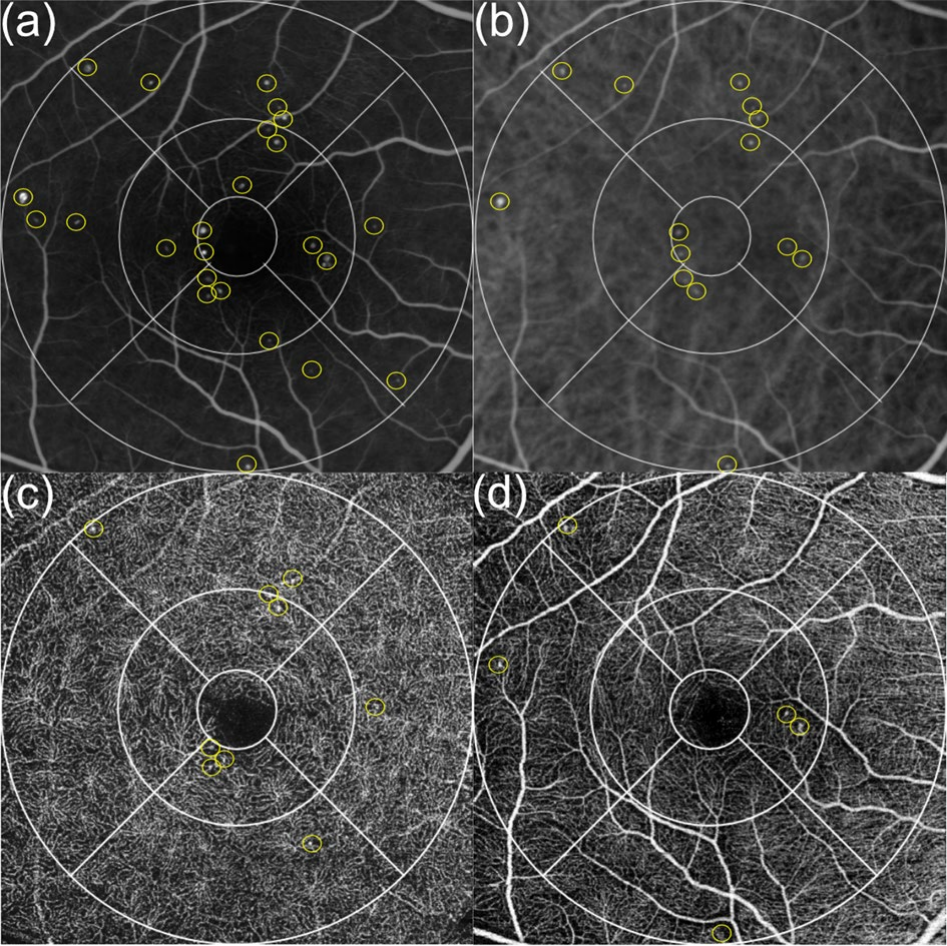
Right eye of a 55-years old woman with mild non-proliferative diabetic retinopathy and diabetic maculopathy. (**a**) Early fluorescein angiography (FA) counting 24 microaneurysms (MA) of different intensity in the total Early Treatment Diabetes Retinopathy Study (ETDRS) grid (**b**) Early indocyanine green angiography presenting 14 macular MAs with similar localization as in FA (**c**) A 6 × 6 mm swept source-optical coherence tomography angiography (SS-OCTA) illustration of the deep capillary plexus (DCP) with 9 definite MAs in the total ETDRS grid (**d**) Five MAs visible in the superficial capillary plexus of the same SS-OCTA cube with 1 MA in the upper left corner of the 6 mm superior sector also visible in the DCP.

ETDRS grids with 1 mm, 3 mm and 6 mm diameter circles in 1-pixel size were drawn by Affinity and built in the 6 × 6 mm borders of the automated SCP and DCP en-face images (Fig. 1). The 3 mm and 6 mm diameter circles were split into the superior, temporal, inferior and nasal sector according to the grid. The SS-OCTA images where superimposed with the en-face angiographic images by an exact alignment of the inner retinal vasculature. All visible MAs in FA, ICGA, SCP and DCP were counted independently in each sector as well as in the total grid. In FA, MAs were counted only if not obscured by leakage. If an MA was well delineated in FA and ICGA, it was counted as one visible in both dye-based angiographies (Fig. 1a, 1b). In SS-OCTA, we anticipated the different morphological appearances of MAs as described in other studies (Fig. 1c, 1d)^21^. In the rare case of grading disagreement between two independent readers beyond 3 MAs within one sector, a senior clinical advisor was consulted. To investigate the interrater agreement, intraclass correlation coefficients (ICC) were calculated separately for the different device settings. Overall, a good ICC > 0.9 could be detected (Table 1). A smaller mean value (0.69) was only found between both graders for the numbers of MAs in the SCP.

**Table 1:** Diabetic macular microaneurysms as means and medians as well as intraclass correlation coefficients for the total ETDRS grid and for each sector in all eyes (n = 27).

	Dye-based angiography		OCTA layers	
	FA	ICGA	SCP	DCP
Total grid mean ± standard deviation median (q1;q3) ICC	33.44 ± 22 27.5 (21.75;38.25) 0.98	24.93 ± 16.87 17.5 (14;35) 0.98	6.48 ± 3.7 5.5 (3.75;9.25) 0.69	18.07 ± 10.47 18.5 (10.75;23.5) 0.92
1 mm	0.98 ± 1.18 0 (0;2) 0.97	0.69 ± 1.06 0 (0;1) 0.95	0.33 ± 0.84 0 (0;0) 0.85	0.59 ± 0.78 0 (0;1) 0.83
3 mm superior	2.26 ± 2.43 2 (1;3.25) 0.95	1.85 ± 1.92 1 (0.25;2.75) 0.93	0.57 ± 0.83 0 (0;1) 0.74	1.87 ± 1.86 1.5 (0.5;2.25) 0.91
3 mm temporal	2.46 ± 2.43 2 (1;3.5) 0.98	1.61 ± 2.11 1 (0;2.5) 0.99	0.54 ± 1 0 (0;0.75) 0.87	1.85 ± 1.67 1.5 (1;2.25) 0.85
3 mm inferior	2.63 ± 2.49 2 (1;3.5) 0.97	1.85 ± 1.73 1.5 (0.75;3) 0.94	0.54 ± 0.82 0 (0;1) 0.77	1.5 ± 1.34 1 (0.5;2) 0.91
3 mm nasal	2 ± 2.02 1.5 (0.25;3) 0.97	1.87 ± 1.96 1 (0.75;2.75) 0.93	0.74 ± 0.71 0.5 (0;1) 0.64	1.11 ± 1.21 0.5 (0;29) 0.79
6 mm superior	6.5 ± 8.56 3 (1;7) 0.99	4.43 ± 5.83 2.5 (1;4.75) 0.99	1 ± 1.18 0.5 (0;2) 0.87	3.37 ± 3.49 2.5 (0.5;4.75) 0.96
6 mm temporal	6.57 ± 6.06 4 (2;9) 0.98	5.33 ± 5.15 4 (1;7.75) 0.98	1.26 ± 1.44 1 (0;2) 0.85	3.56 ± 3.26 3 (1;4.5) 0.93
6 mm inferior	5.52 ± 4.54 4 (1.75;7.75) 0.97	3.78 ± 4.02 2.5 (1;4.75) 0.98	0.61 ± 0.8 0 (0;1) 0.81	2.39 ± 2.16 2 (1;3.25) 0.93
6 mm nasal	4.31 ± 5.62 2.5 (1;5.75) 0.98	3.56 ± 4.28 2 (0.5;5.25) 0.97	0.89 ± 0.92 1 (0;1) 0.8	1.76 ± 2.14 1 (0.25;2.5) 0.97

*ETDRS* early treatment diabetes retinopathy study, *OCTA* optical coherence tomography angiography, *FA* fluorescein angiography, *ICGA* indocyanine green angiography, *SCP* superficial capillary plexus, *DCP* deep capillary plexus, *q1* quartile 1, *q3* quartile 3, *ICC* intraclass correlation coefficient.

### Quantitative MA analysis based on imaging technique and localization

**Figure 2:**
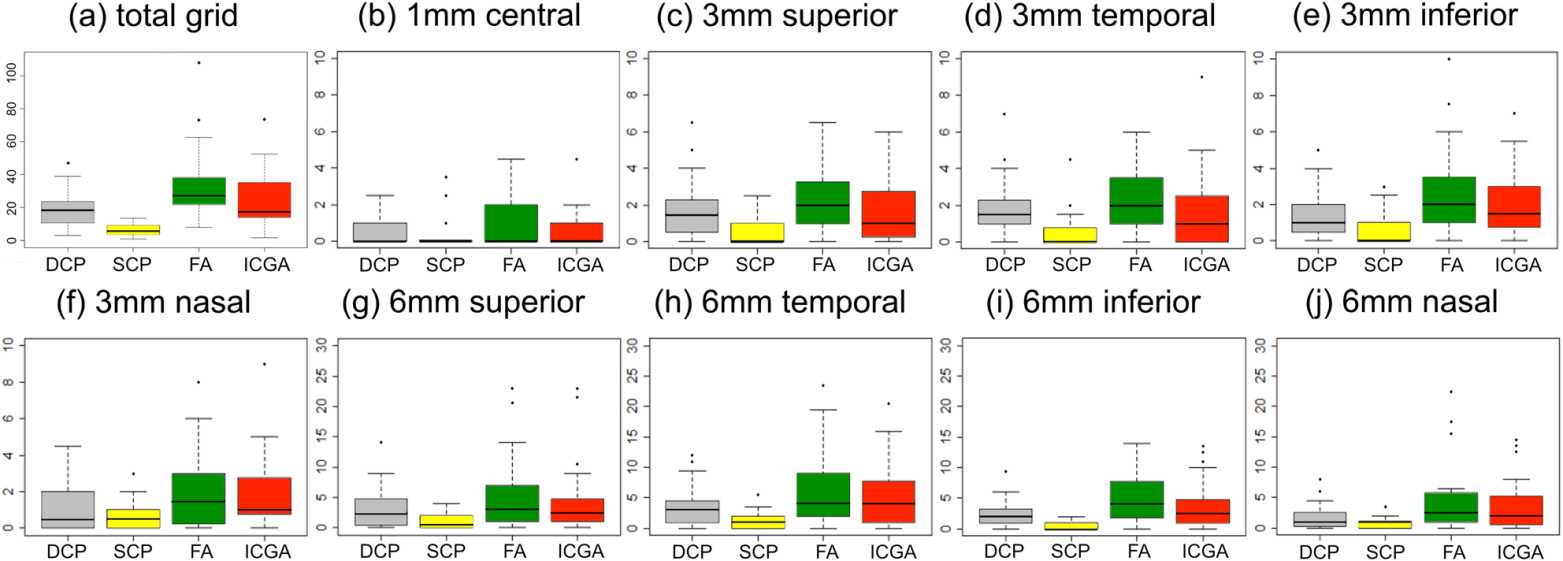
Numbers and location of diabetic macular microaneurysms (MA). For each color box-and-whisker plot, the horizontal bar within the box represents median; top and bottom of box, interquartile range. Upper whisker extends from the upper quartile to the closest observed data point below the upper quartile plus 1.5 times the interquartile range; lower whisker extends from the lower quartile to the closest observed data point above the lower quartile minus 1.5 times the interquartile range. Outlying values are plotted as dots. (**a**) The total Early Treatment Diabetes Retinopathy Study grid and for (**b–j**) every sector independently. The deep capillary plexus (DCP = grey) of the swept source-optical coherence tomography angiography (SS-OCTA) and indocyanine green angiography (ICGA = red) showed similar medians in the total as well as in most sectors. The superficial capillary plexus (SCP = yellow) of SS-OCTA counted least MAs, while fluorescein angiography (FA = green) demonstrated the highest numbers overall independent of the sector.

**Figure 3:**
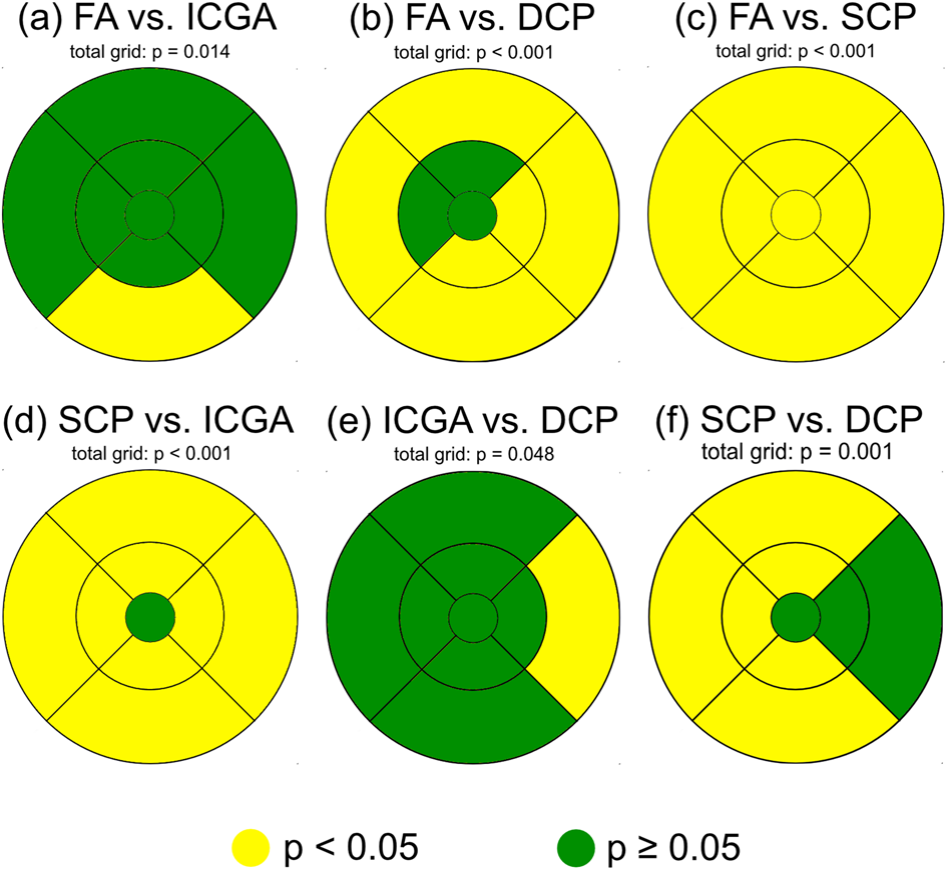
Color map of significance levels (*p* < 0.05 = yellow, *p* ≥ 0.05 = green) in the total Early Treatment Diabetes Retinopathy Study grid and for each sector comparing **(a)** fluorescein angiography (FA) to indocyanine green angiography (ICGA), **(b)** FA to the deep capillary plexus (DCP), **(c)** FA to the superficial capillary plexus (SCP) **(d)** the SCP to ICGA, **(e)** ICGA to the DCP and **(f)** the SCP to the DCP. All eyes were displayed as right eyes representative for both lateralities (86% right and 14% left eyes).

The total numbers of MAs were 33.44 ± 22 (standard deviation) (median 27.5 [q1:21.75;q3:38.25]) on average in FA, 24.93 ± 16.87 (17.5 [14;35]) in ICGA, 6.48 ± 3.7 (5.5 [3.75;9.25]) in the SCP and 18.07 ± 10.47 (18.5 [10.75;23.5]) in the DCP. A mean of 21.3 ± 14.2 (17.5 [13;27]) MAs were visible in both dye-based angiograms, while 4.98 ± 3.1 (5 [2.25;7.25]) MAs where present in both SS-OCTA layers. A detailed list of each imaging modality and ETDRS grid sector was displayed in Table 1. Box plot diagrams and whiskers with median, 25 and 75 percentiles as well as range and outlying values were displayed in total and for each ETDRS sector as shown in Fig. 2. *P* values (mixed effects models including 95% confidence intervals [95% CI]) between all imaging modalities for the total grid as well as for each sector were listed in Table 2. A color map for each comparison and localization was displayed in Fig. 3 in order to ease visualization of significance levels (*p* < 0.05). A statistically significant difference between FA and ICGA could be observed only in the inferior sector of the 6 mm diameter (Fig. 3a). FA and DCP demonstrated non-significance in the 1 mm center, the 3 mm superior and 3 mm temporal sector, while all other quadrants were statistically significantly different (Fig. 3b). FA and SCP were statistically different in all sectors including the 1 mm center (Fig. 3c). SCP versus ICGA showed statistically significant differences in all but the central sector (Fig. 3d). When comparing ICGA to DCP, non-significant results were predominant with borderline significant levels in the nasal sector of the inner 3 mm diameter, the 6 mm temporal, 6 mm inferior and 6 mm nasal sector as well as the total number of MAs (Fig. 3e). Comparing the MA numbers displayed in the SCP and the DCP, the difference was statistically significant in all but the central, the 3 mm nasal and the 6 mm nasal sector (Fig. 3f).

**Table 2:** Mean difference of diabetic macular microaneurysms comparing each imaging modality for the total ETDRS grid and for each sector in all eyes (n = 27).

	FA versus ICGA	FA versus DCP	FA versus SCP	SCP versus ICGA	ICGA versus DCP	SCP versus DCP
Total grid *p* value (95% CI)	8.52 *p* = 0.014 (1.88;15.16)	15.37 *p* < 0.001 (8.73;22.01)	26.96 *p* < 0.001 (20.32;33.6)	− 18.44 *p* < 0.001 (− 25.08;− 11.81)	6.85 *p* = 0.048 (0.21;13.49)	− 11.59 *p* < 0.001 (− 18.23;− 4.95)
1 mm	0.2 *p* = 0.343 (− 0.21;0.62)	0.3 *p* = 0.169 (− 0.12;0,71)	0.56 *p* = 0.011 (0.14;0.94)	− 0.35 *p* = 0.103 (− 0.77;0.06)	0.09 *p* = 0.666 (− 0.32;0.51)	− 0.26 *p* = 0.228 (− 0.68;0.16)
3 mm superior	0.41 *p* = 0.282 (− 0.33;1.14)	0.39 *p* = 0.305 (− 0.34;1.12)	1.69 *p* < 0.001 (0.95;2.42)	− 1.28 *p* = 0.001 (− 2.01;− 0.54)	− 0.02 *p* = 0.961 (− 0.75;0.72)	− 1.3 *p* = 0.001 (− 2.03;− 0.56)
3 mm temporal	0.85 *p* = 0.058 (− 0.01;1.72)	0.61 *p* = 0.172 (− 0.25;1.48)	1.93 *p* < 0.001 (1.06;2.79)	− 1.07 *p* = 0.018 (− 1.94;− 0.21)	− 0.24 *p* = 0.589 (− 1.11;0.62)	− 1.31 *p* = 0.004 (− 2.18;− 0.45)
3 mm inferior	0.78 *p* = 0.735 (− 0.02;1.57)	1.13 *p* = 0.007 (0.33;1.93)	2.09 *p* < 0.001 (1.3;2.89)	− 1.31 *p* = 0.002 (− 2.11;− 0.52)	0.35 *p* = 0.392 (− 0.44;1.15)	− 0.96 *p* = 0.021 (− 1.26;− 0.17)
3 mm nasal	0.13 *p* = 0.735 (− 0.61;0.87)	0.89 *p* = 0.022 (0.15;1.63)	1.26 *p* = 0.001 (0.52;2)	− 1.13 *p* = 0.004 (− 1.87;− 0.39)	0.76 *p* = 0.05 (0.02;1.5)	− 0.37 *p* = 0.335 (− 1.11;0.37)
6 mm superior	2.07 *p* = 0.082 (− 0.22;4.37)	3.13 *p* = 0.009 (0.84;5.42)	5.5 *p* < 0.001 (3.21;7.79)	− 3.43 *p* = 0.005 (− 5.72;− 1.13)	1.06 *p* = 0.372 (− 1.24;3.35)	− 2.37 *p* = 0.047 (− 4.66;− 0.08)
6 mm temporal	1.24 *p* = 0.168 (− 0.5;2.98)	3.02 *p* = 0.001 (1.28;4.76)	5.31 *p* < 0.001 (3.58;7.05)	− 4.07 *p* < 0.001 (− 5.81;− 2.34)	1.78 *p* = 0.05 (0.04;3.52)	− 2.3 *p* = 0.012 (− 4.03;− 0.56)
6 mm inferior	1.74 *p* = 0.017 (0.35;3.13)	3.13 *p* < 0.001 (1.74;4.52)	4.91 *p* < 0.001 (3.52;6.3)	− 3.17 *p* < 0.001 (− 4.56;− 1.78)	1.39 *p* = 0.055 (0;2.78)	− 1.78 *p* = 0.015 (− 3.17;− 0.39)
6 mm nasal	0.76 *p* = 0.385 (− 0.94;2.45)	2.56 *p* = 0.004 (0.86;4.25)	3.43 *p* < 0.001 (1.73;5.12)	− 2.67 *p* = 0.003 (− 4.36;− 0.97)	1.8 *p* = 0.042 (0.1;3.49)	− 0.87 *p* = 0.32 (− 2.57;0.82)

*ETDRS* early treatment diabetes retinopathy study, *FA* fluorescence angiography, *ICGA* indocyanine green angiography, *DCP* deep capillary plexus, *SCP* superficial capillary plexus, *95% CI* confidence interval.

## Discussion

This prospective explorative study was the first to detect a plausible agreement in the quantitative appearance of clinically relevant diabetic macular MAs when comparing dye-based angiographies to each other and to the non-invasive SS-OCTA technology split into the anatomically diverse SCP and DCP, let all morphological descriptions of MAs in OCTA—including the absent type—aside^21^. FA showed the highest number of MAs in total as well as in each quadrant, followed by ICGA and—closely—the DCP. The mean MA numbers in FA and ICGA were different (*p* = 0.014 [1.88;15.16]), although only one sector showed a significant value when analyzed independently. The median was more useful in this setting than the mean due to the high numbers of MAs in only a few quadrants, also graphically illustrated by the box plot charts and their spikes (Fig. 2). Hwang et al. highlighted the limits of extensive leakage in FA obscuring not only capillary dropout but also mimicking MAs in contrast to OCTA, which revealed them to be small neovascularizations protruding vertically above the inner limiting membrane^22^. Ogura and colleagues superimposed FA and ICGA images of MAs to guide focal laser treatment for macular edema. They adjudged ICGA to be helpful in finding leaking spots in CSME and probably even in diffuse edema^23^. Farias et al. recently investigated the feasibility of ICGA in DR and distinguished between MAs smaller than 150 µm and telangiectatic capillaries^24^. They concluded that early phases were more helpful for the detection of MAs while later staining revealed larger telangiectatic vessels. In accordance with their findings we considered early ICGA immensely helpful in the detection and quantification of MAs. In a recent Japanese study, all available imaging technologies including ICGA and OCT B-scans were performed, where highly luminescent ring-shaped morphologies were considered to be MAs and thus detected vertically in the deeper rather than the superficial layers^25^. Their results conformed with our numbers in regard of the topographical localization in the deep plexus and of the lower detection rate in ICGA compared to FA. We can only hypothecate the reasons for that but believe that ICGA has implications as a valuable grading tool for the quantitative assessment of MAs in the deeper retina based on its characteristics. Thus said, the negative attributes of dye-dependent angiography remain but give way to the era of non-invasive, highly reproducible and depth-resolved OCTA.

In our study, least MAs in SS-OCTA were found in the SCP from the inner limiting membrane (ILM) to the inner plexiforme layer (IPL), while the majority was situated in the DCP between the IPL and the outer plexiforme layer (OPL). These results conform to earlier studies on the quantitative appearance of MAs preliminary found in the deeper segmentations of SS-OCTA^26^. Querques and his colleagues performed simultaneous FA and SD-OCT B-scans in 11 diabetic patients with DR to some degree and found abnormal capillaropathy interpreted as MAs in the deep capillary network even extending into the outer nuclear layer^27^. Jia et al. investigated a case of DR and alluded to its future domain in the central 3 mm and 6 mm zones as used in ETDRS^28^. We were mostly interested in the juxta- and perifoveal region as a source of CSME. Ishibizawa et al. used a commercially available SD-OCTA for investigating all kinds of diabetic vascular abnormalities^29^. They discovered morphological patterns of MAs such as saccular or fusiform and assigned them to different retinal layers—namely the SCP and the DCP. Soares et al. compared MA counts in FA to the central 3 mm zones of 2 SD-OCTA devices and found less MAs in the superficial layer as well as in the full retina slab in both^16^. Couturier and her colleagues detected differences in plexus anomalies dependent on the location and saw more MAs in the DCP rather than the SCP^17^. Another paper described an overall of 77% of MAs detected in the DCP increasing to 91% if the diabetic edema was thicker than 400 µm^18^. The numbers of MAs present in both plexi were comparable to the numbers in the SCP alone. The meaning of this phenomenon is twofold: Some MAs could be projections from the superficial plexus onto the deeper layers. If so, it would falsely increase the numbers counted in the DCP. On the other hand, we detected far more MAs in the DCP than in the SCP and used the integrated artefact removal option. It would be conclusive that some MAs from the DCP were additionally visualized in the SCP, either caused by a disintegration of the retinal architecture in edema or simply due to their size or flow pattern as pictured by the automated segmentation. MAs were counted in both layers if it was not possible to assign them to the respective plexus. Peres et al. found more MAs in the DCP than in FA in 19 diabetic eyes^19^. Schaal et al. used SS-OCTA central and wide field (12 × 12 mm) images for grading DR and correlated them with color fundus photography as used in ETDRS^30^. They summed up all vascular plexus and found MAs present in 91% of cases in contrast to fundus photography. As a conclusion, they suggested SS-OCTA as a screening tool even in early DR as it might be more sensitive than presumed.

The median numbers in ICGA and the DCP of SS-OCTA resembled each other in most sectors of the ETDRS grid (Fig. 2) while only a few mean numbers showed borderline significances and therefore gained most of our attention. Kaizu et al. investigated the MA detection rate with 2 SD-OCTA based devices and compared it to ICGA^31^. They averaged the images up to ten times but could not find a correlation with either averaging time or ICG angiograms. Their work showed a significant correlation between different morphological characteristics—particularly the bulge types—and visibility. MAs must have the capability to accumulate dye or ascertain flow to be detected by one of the technologies. Although we did not investigate morphology separately, only MAs with dye staining or flow signal in SS-OCTA were eligible for enrollment. We meticulously counted hyperfluorescent dots in dye-based angiography as well as MA subtypes in the respective plexus of SS-OCTA and found good agreement in numbers between ICGA and the DCP with a preponderance towards depth-independent ICGA.

Major limitations of this research include its low numbers. This is partially attributed to the explorative character despite its comparative nature. ICGA is associated with dye-dependent risk factors but not a first line diagnostic tool for graduating diabetic maculopathy. Therefore, it was not integrated into our clinical routine. Due to the lack in numbers we could not distinguish between cubes consisting of 320 or 512 B-scans, although a higher scan density would implement a better resolution and hence should lead to a more accurate detection rate. Also, eyes with and without CSME as well as NPDR and PDR were included as long as the macular region was not endangered by tractive detachment or obscuration by blood. Edema is expressed as early leakage in FA but could also lead to a miscount of MAs in other imaging techniques^13,16,17,21,32^. A more homogenous collective would emphasize the clarity of our data. Grading was performed by two readers, while another advisor was only consulted in the rare incidence of disagreement above 3 MAs. ICC was excellent for all but the SCP as listed in Table 1, which left a possible grading bias but could likely be owed to the low numbers in this segmentation. On the other hand, this is the first explorative prospective study which compared ICGA to SS-OCTA with emphasis to the DCP.

To conclude, ICGA showed similar outcome values compared to the DCP in SS-OCTA regarding the detection rate of clinically relevant diabetic macular MAs. We believe that our results contribute to the imaging methodology in DM.

## Methods

### Ethical approval and study participants

The clinical trial with the registration number EK-17-083-0517 was approved by the Viennese ethical commission and adhered to the tenets of the Declaration of Helsinki. Informed consent was obtained from voluntary participants after they had been informed about the aims of the study, the procedures involved, their potential present and future benefit as well as possible risks.

### Study protocol

All examinations in this prospective explorative observational study took place between September 2018 and August 2019. Indirect slit-lamp biomicroscopy (Haag-Streit AG, Bern, Switzerland) with dilated pupils using 0.5% tropicamide (MYDRIATICUM, Agepha Pharmaceuticals, Vienna, Austria) drops, multimodal imaging including spectral domain (SD)-OCT (Zeiss Cirrus HD 4000, Carl Zeiss Meditec AG, Jena, Germany) and FA were performed as standard of care for the detection of DM but immediately extended with ICGA (SPECTRALIS HRA-OCT Confocal Scanning Laser Ophthalmoscope and Angiography; Heidelberg Engineering, Heidelberg, Germany). In addition, the central retina was scanned with Topcon’s DRI-OCT Triton Plus (Topcon Corporation, Tokyo, Japan) device using SS-OCTA consecutively on the same day. Heidelberg’s SPECTRALIS scanning laser ophthalmoscope provides 30° en-face images with an isotropic transversal resolution between 11 and 40 µm in high-speed settings independent of the administered dye or laser diode and is equipped with an eye-tracker. In each patient, both eyes were examined parallel with a series of consecutive pictures starting approximately at 30 s after dye injection with dilated pupils. The distribution of dye changed over time and images were recorded up to 15 min after dye injection. Early FA pictures were hence investigated in each eye for comparability due to severe leakage in later phases (Fig. 1a). Early ICGA phases were preferred as later phases typically demonstrated wash-out of retinal vasculature and sometimes MAs (Fig. 1b). Consenting patients were additionally investigated by a beta-version of Topcon’s DRI-OCT Triton Plus with SS-OCTA by an experienced operator under mesopic lighting conditions on the same day. This swept source device operates on 1 mW input power and preserves axial resolution by using a full-spectrum motion contrast algorithm to create a decorrelation signal, called OCTARA (OCTA Ratio Analysis). It is equipped with an active eye tracker for reducing motion artefacts as well as a projection artefact removal algorithm. All acquired scans were captured in “low eye-tracker” option to reduce examination time.

### Image analysis and grading

Topcon’s integrated OCTA analysis software IMAGEnet 6 (Version 1.24.1.15742, Tokyo, Topcon Corporation, Tokyo, Japan) provides 3 × 3 mm, 4.5 × 4.5 mm, 6 × 6 mm, and even 9 × 9 mm macular cubes with automated subdivision into four different en-face layers: the SCP, the DCP, the outer retina and the choriocapillaris. Topcon’s device does not come with an automated segmentation of the ICP and therefore this interesting analysis could not be performed. Standard 6 × 6 mm cubes with a volume of 320 B-scans or higher resolution 512 B-scan cubes were captured. While the digital axial resolution was 2.6 µm, the transverse resolution ranged from 9.4 to 18.8 µm depending on the selected cube. The integrated software was used to delineate the SCP and DCP automatically. The SCP was defined as a slab between 2 lines drawn from the ILM to the outer limits of the IPL (Fig. 1c) followed subsequently by the DCP layer from the outer limits of the IPL to the outer limits of the OPL (Fig. 1d). Semi-automated segmentation meaning a manual shift of the predefined lines or complete manual re-segmentation was avoided to reduce the influence of the grader and hence obscuration of the data. Images with artefacts affecting the quantification of MAs in one of the segmentations either by obscuration (typically white or black bands in case of motion) or segmentation errors due to severe retinal distortion were excluded. Further image post processing for quality enhancement was not performed in order to identify raw data material.

### Statistical analysis

Continuous variables were summarized using mean, standard deviation, median, quartiles as well as minimum and maximum. Categorical variables were summarized using absolute and percent values. Mixed effects models with random factor patient were calculated to investigate influencing factors on the corresponding device value. It accounts for the fact that all imaging techniques were used within each patient and compared to each other. For these models, the mean value over both graders was used.

No correction for multiplicity was performed due to the exploratory character of the study. The global statistical significance level was set to 0.05 and *p* values < 0.05 were considered as statistically significant. The mean bias (95% CI) was calculated for main findings. All analyses were performed using R, release 3.3.3. Diagrams were illustrated by using SPSS 20 (IBM Corporation, New York, USA). Figures were composed utilizing Affinity Photo 1.7 (Serif Europe Ltd, Nottingham, UK).

## Data Availability

Martin Stattin and Siamak Ansari Shahrezaei had full access to all the data in the study and take responsibility for the integrity of the data and the accuracy of the data analysis. The datasets used and/or analyzed to support the findings of this study are available from the corresponding author on reasonable request.
